# Distinctive visual tasks for characterizing mild cognitive impairment and dementia using oculomotor behavior

**DOI:** 10.3389/fnagi.2023.1125651

**Published:** 2023-07-20

**Authors:** Dharma Rane, Deba Prasad Dash, Alakananda Dutt, Anirban Dutta, Abhijit Das, Uttama Lahiri

**Affiliations:** ^1^Indian Institute of Technology Gandhinagar, Electrical Engineering, Palaj, Gujarat, India; ^2^AMRI Hospital, Mukundapur, Kolkata, West Bengal, India; ^3^Jacobs School of Medicine and Biomedical Sciences, University at Buffalo SUNY, Buffalo, NY, United States; ^4^Walton Centre NHS Foundation Trust, Liverpool, United Kingdom

**Keywords:** dementia, eye tracking, mild cognitive impairment, saccade, spatial memory

## Abstract

**Introduction:**

One’s eye movement (in response to visual tasks) provides a unique window into the cognitive processes and higher-order cognitive functions that become adversely affected in cases with cognitive decline, such as those mild cognitive impairment (MCI) and dementia. MCI is a transitional stage between normal aging and dementia.

**Methods:**

In the current work, we have focused on identifying visual tasks (such as horizontal and vertical Pro-saccade, Anti-saccade and Memory Guided Fixation tasks) that can differentiate individuals with MCI and dementia from their cognitively unimpaired healthy aging counterparts based on oculomotor Performance indices. In an attempt to identify the optimal combination of visual tasks that can be used to differentiate the participant groups, clustering was performed using the oculomotor Performance indices.

**Results:**

Results of our study with a group of 60 cognitively unimpaired healthy aging individuals, a group with 60 individuals with MCI and a group with 60 individuals with dementia indicate that the horizontal and vertical Anti-saccade tasks provided the optimal combination that could differentiate individuals with MCI and dementia from their cognitively unimpaired healthy aging counterparts with clustering accuracy of ∼92% based on the saccade latencies. Also, the saccade latencies during both of these Anti-saccade tasks were found to strongly correlate with the Neuropsychological test scores.

**Discussion:**

This suggests that the Anti-saccade tasks can hold promise in clinical practice for professionals working with individuals with MCI and dementia.

## Introduction

Globally, dementia is the third most serious health problem following cancer and cardio-cerebrovascular diseases (Martin et al., 2016). Despite lacking effective treatment once established, early multidomain intervention measures are emerging as effective tools for managing dementia (Ngandu et al., 2015), if implemented before dementia onset. Mild cognitive impairment (MCI) has been conceptualized as an intermediate phase between normal cognitive aging and overt dementia (Geda, 2012). MCI is characterized by subtle deficits in memory and/or other cognitive domains (usually to a less severe extent than dementia and without significant impairment in activities of daily living) that can be captured using biomarkers in response to distinctive visual tasks (Anderson and MacAskill, 2013). One such biomarker includes eye movements during visual tasks. This is because one’s eye movement provides a unique window into the cognitive processes (Wedel et al., 2023) and higher-order cognitive functions (e.g., memory) (Anderson and MacAskill, 2013; Molitor et al., 2015).

One’s eye movement can be captured by eye tracking techniques. Given the promise of eye tracking, it has been used by researchers working with individuals with dementia and MCI while taking part in a visual task that offered dynamic and static stimuli (Oyama et al., 2019). Also, researchers have investigated oculomotor behavior (in terms of eye fixation) of individuals with healthy aging, MCI and those with dementia while using different visual stimuli (Oyama et al., 2019; Nie et al., 2020). Researchers have used Pro-saccade, Anti-saccade and Memory-based fixation tasks while exploring oculomotor behavior of individuals with Alzheimer’s Disease and Fronto-temporal Dementia (Lage et al., 2021). The reason behind using such visual tasks is that saccades (requiring bottom-up control (Takacs and Wechsler, 1996) toward a target stimulus (i.e., Pro-saccades) presented on either side of a central fixation point often show prolonged latencies (inferring increased reaction time) for those with cognitive impairment and having bottom-up control as adversely affected (Takacs and Wechsler, 1996; Zhang et al., 2015) than their cognitively unimpaired counterparts. Also, task-specific fixation on spatially distributed target locations while testing spatial working memory, such as in Memory Guided fixation tasks (Chan et al., 2005) can offer information on one’s cognitive impairment (Rosse et al., 1992). Additionally, saccades in the opposite direction [requiring top-down control (Munoz and Everling, 2004) to a visual target (i.e., Anti-saccades) have been shown to elicit pronounced effect on the saccade latency possibly due to top-down control being adversely affected in those with cognitive impairment (van Stockum et al., 2011; Zhang et al., 2015)]. The Anti-saccade task requiring higher-level voluntary control, involving extensive cortical areas (Hallett, 1978) and needing inhibitory control (Hutton, 2008) that is fueled by the top-down control (van Stockum et al., 2011) have been reported as powerful while investigating eye movement of individuals with MCI (Heuer et al., 2013; Opwonya et al., 2022) and dementia (Meyniel et al., 2005; Russell et al., 2021). In addition, saccade direction has also been investigated. Specifically, researchers have reported that individuals with cognitive decline face difficulty in shifting gaze in the vertical direction (Hotson and Steinke, 1988) that is true for both tasks requiring reflexive and inhibitory saccades. Thus, researchers have been focusing on investigating saccades in the vertical direction and Anti-saccades while studying the oculomotor behavior of individuals with Parkinson’s Disease, dementia etc., (Hotson and Steinke, 1988; Waldthaler et al., 2019).

Given the possibility of applying one’s oculomotor behavior (in response to various visual tasks) in characterizing MCI and dementia and the importance of Anti-saccade tasks (requiring inhibitory control) and tasks requiring vertical saccades (both being likely to be adversely affected in MCI and dementia), we hypothesize that Anti-saccade task and vertical saccade tasks can be used to differentiate individuals with MCI and dementia from their cognitively unimpaired healthy aging counterparts based on their task-specific oculomotor behavior. As a step toward achieving this, our first aim was to understand the visual task-specific oculomotor behavior as captured by an eye tracking setup (quantified in terms of oculomotor Performance indices) of a group of cognitively unimpaired healthy aging individuals, a group with MCI and a group with dementia labeled using standard Neuropsychological tests (that needs to be administered by trained resources). Our second aim was to understand the relation between the visual task-specific oculomotor Performance indices with the Neuropsychological test scores. Our third aim was to identify the optimal combination of visual tasks that can be used to cluster the participant groups based on the oculomotor Performance indices. Finally, we wanted to ensure that the variations in oculomotor behavior (in response to the visual tasks) were not due to participants’ demographics.

## Materials and methods

### System design

The gaze-sensitive platform comprised of (a) eye tracking and (b) computer-based visual task modules.

### Eye tracking module

The eye tracking module that we used comprised of Intel Realsense SR300 camera. We used the infrared (IR) camera mode that allows for video capture at 200 fps camera (Intel, 2016) instead of the depth-sensing camera mode that offers lower frame rate. The Intel librealsense API (OpenCV) was used to extract one’s 2D gaze coordinates (Patel et al., 2017) corresponding to a visual stimulus [discussed below; presented on the monitor (1,280 × 1,024 pixels)] of a Task Computer along with time stamping.

One’s gaze data was processed to remove noise due to blinks and invalid fixations [lying outside the monitor and lasting for ≤50 ms (Nyström and Holmqvist, 2010)]. For detection of saccade, we considered gaze velocity lying within 300°/s and 1,000°/s (Munoz et al., 2003) and the gaze data was monotonic within each 50 ms window with the difference between the maximum and minimum values of gaze coordinates (within the selected window) being >2 (Polden et al., 2020).

### Computer-based visual tasks

The gaze-sensitive platform offered visual stimuli in the form of (1) Pro-saccade, (2) Anti-saccade, and (3) Memory-Guided Fixation tasks (described below) projecting Interim Refresh Screen (Screen_IR_ henceforth), Preparatory Screen (Screen_P_ henceforth) and Stimulus Screen (Screen_S_ henceforth) with the Screen_S_ varying based on the visual task. The Memory Guided Fixation task projected an additional Fixation Screen (Screen_*Fix*_ henceforth). The Screen_IR_ was a white-colored blank screen presented on the monitor. The Screen_P_ was a white-colored screen displaying a central fixation point (CFP henceforth; “ + ”; 0.65° × 0.65° in size). The Screen_S_ was a white-colored screen displaying a CFP and target stimulus [Target henceforth; black colored circle 1.25° in size with a tiny central white dot of 0.25° (looking from 50 cm)]. These dimensions were chosen based on pilot trial with individuals, age-matched with our participant pool. For Pro-saccade and Anti-saccade tasks, we used the overlap method (where the CFP stays on the screen after Target onset) instead of the gap method (in which the CFP disappears prior to Target onset) (Intel, 2016). This is because, the overlap method has been reported to lead to a more pronounced manifestation of gaze behavior measured in terms of saccade latency (Polden et al., 2020).

### Pro-saccade task

**FIGURE 1:**
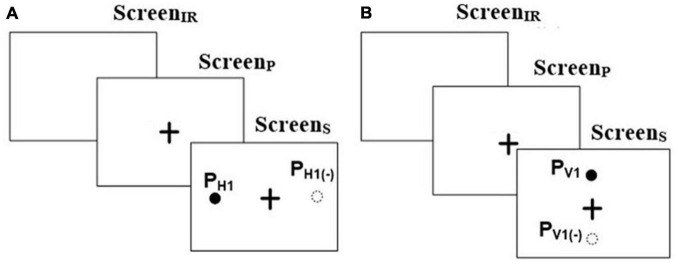
Stimuli for **(A)** Horizontal and **(B)** Vertical Pro-saccade and Anti-saccade. “ + ” in the figure represents the central fixation point.

The Pro-saccade task was one in which a user was expected to look to a visual target on the screen once it appears. This task was of two types, namely, Horizontal Pro-saccade (HPS henceforth) and Vertical Pro-saccade (VPS henceforth). For both the tasks, the Screen_IR_ appeared for 250 ms [minimum blink duration being ∼200 ms (Yang et al., 2013) to facilitate triggering of saccade by an exogenous removal of the fixation activity prior to saccade onset (Yang et al., 2013)] followed by Screen_P_ appearing for 2,000 ms that in turn was followed by Screen_S_ (presenting the Target) for 2,000 ms, similar to that reported in literature (Szűcs et al., 2013). For HPS (Figure 1A), we programed the Target to appear randomly at ± 15° and ± 10° visual angles on the left and right sides of the CFP. For VPS (Figure 1B), the Target was randomly presented at ± 7° on the upper and lower side of the CFP. Specifically, these locations (namely ± 15° and ± 10° in the case of horizontal saccades and ± 7° in the of vertical saccades) for the Target were chosen as a typical case keeping in mind the Field of View of the eye tracking camera setup. The Pro-saccade task required one to shift his/her gaze toward the Target, followed by a fixation on the Target (Yang et al., 2013). For example, for HPS or VPS task, if the Target appeared at P_H1_ or P_V1_ (Figures 1A, B), then the task required one to fixate at P_H1_ or P_V1_, respectively. The gaze-sensitive platform was programed to offer 8 trials for HPS and 4 trials for VPS with 2 trials for each Target location.

### Anti-saccade task

The Anti-saccade task was one in which a user was expected to look in a direction opposite to the visual target appearing on the screen. This task was of two types, e.g., Horizontal Anti-saccade (HAS henceforth) and Vertical Anti-saccade (VAS henceforth). The task presentation was similar to HPS and VPS tasks, respectively. The gaze-sensitive platform was programed to offer 8 trials for HAS and 4 trials for VAS tasks with 2 trials for each Target. The Anti-saccade task required one to perform a relevant saccade i.e., had to shift his/her gaze exactly in the opposite direction to that in which a Target appeared in the visual field (Oyama et al., 2019) while not considering the point of gaze fixation (following the saccade). For example, for HAS or VAS, if the Target appeared at P_H1_ or P_V1_, then the task required one to fixate at P_H1(–)_ or P_V1(–)_, respectively (Figures 1A, B).

### Memory-guided fixation task

**FIGURE 2:**
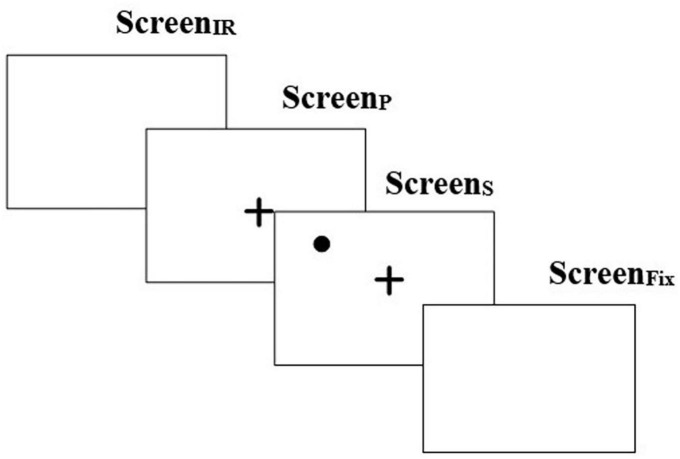
Task stimulus for memory guided fixation task. “ + ” in the figure represents the central fixation point.

The Memory Guided Fixation (MGF) task (Figure 2) was one in which a user was expected to look to a remembered location on the screen after it disappears. This consisted of a Screen_IR_ that appeared for 250 ms followed by Screen_P_ for 2,000 ms which in turn was followed by Screen_S_ (presenting the Target) for 2,000 ms (similar to that used as above). Finally, this was followed by Screen_*Fix*_ that appeared for 2,000 ms. The Screen_*Fix*_ was used to capture whether one could remember the spatial location of the Target (appearing on Screen_S_) and fixate at that location (Brown et al., 2004). The position of the Target was randomly chosen for each quadrant at one of the three preset locations spanning a range of approximately 5° to 11° from the CFP (Nyström and Holmqvist, 2010). The gaze-sensitive platform offered 8 trials of MGF task.

## Methodology

### Participants

Participants (*n* = 180; Table 1) were recruited from a community center having a cohort of individuals coming from divergent educational backgrounds. Data collection site was in the eastern part of India wherein studies have reported prevalence of dementia even in individuals aged in the 50s (Das et al., 2012; Dhiman et al., 2021). Additionally, given that for a clinical syndrome such as dementia, no single definitive test exists, the classification of the participants into three groups, e.g., cognitively Healthy aging Controls (HC henceforth), MCI, and D was done by a panel of expert clinicians. This was in line with the well-accepted clinical practice in which many clinical researchers rely on a process of data review, adjudication, and consensus by a panel of expert clinicians (Weir et al., 2011; Lee et al., 2023). This panel of practicing clinicians evaluated the participants based on thorough evaluations including extensive histories from the participant and an informant, medical history, report of functional abilities, any change in cognition recognized by the affected individual or the informant while considering specific criteria. For example, the criteria for MCI was report on cognitive complaint (beyond what is felt normal for age) but relatively intact functional abilities, that for D was report on memory impairment, aphasia and/or apraxia, or an impairment in executive function and that for HC were absence of active neurological or psychiatric disease, psychotropic medications, or medical disorders (or treatment) compromising cognitive function. This panel of experienced clinicians used their clinical judgment while combining the subjective evaluations with Neuropsychological scoring [similar to that in literature (Petersen, 2004)] obtained using paper-and-pencil based scoring, such as Addenbrooke Cognitive Examination [ACE-III; (Senda et al., 2020)] and Montreal Cognitive Assessment [MoCA (Nasreddine et al., 2005)] (details in Neuropsychological scoring used subsection) for classifying the participants into HC, MCI, and D groups. With regard to education, on an average, ∼38% of the participants had “Primary” education (with a distribution of ∼1:1.1:1.3 belonging to HC, MCI, and D groups, respectively), ∼49% had attended “High School” (with a distribution of ∼1.1:1.1:1 belonging to HC, MCI, and D groups, respectively) and the remaining ∼13% had attended “Junior college and above” (with a distribution of ∼1.2:1.2:1 belonging to HC, MCI, and D groups, respectively). The inclusion criteria were (i) age between 40 and 90 years, (ii) able to give informed consent, (iii) see computer screen 50 cm apart, (iv) follow instructions and (v) participate in paper-and-pencil based scoring. Those with recent eye-related surgery were excluded. Our study followed institute ethics.

**TABLE 1:** Participants’ characteristics.

Participant group	Number of participants	Age [Mean (SD) in years]	ACE-III [Mean (SD) score]
HC	60 (m:50; f:10)	54.41 ( ± 7.89)	88.56 ( ± 3.7)
MCI	60 (m:48; f:12)	57.12 ( ± 8.29)	71.5 ( ± 5.9)
D	60 (m:33; f:27)	57.64 ( ± 10.41)	49 ( ± 7.92)

### Experimental setup

**FIGURE 3:**
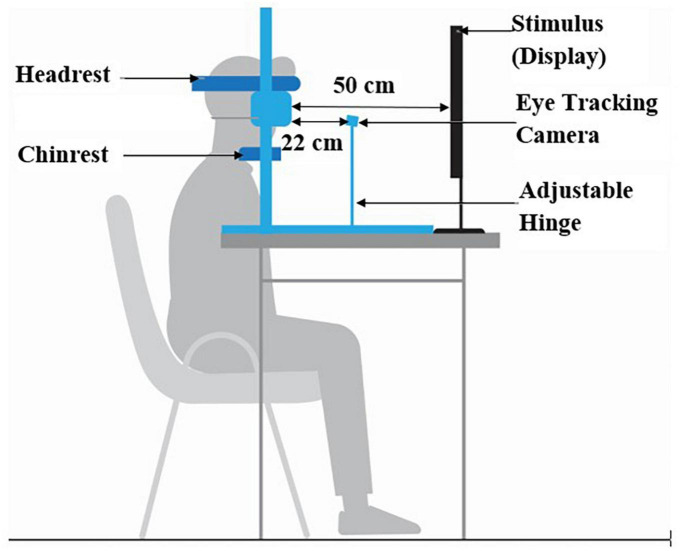
Gaze-sensitive task platform setup.

The experimental setup comprised of (i) chin-rest, (ii) Task Computer and (iii) eye tracking module (Figure 3). An in-house built height-adjustable chin-rest along with head-rest was used. The Task Computer was used to project the visual stimuli for the HPS, VPS, HAS, VAS, and MGF tasks. The study room was uniformly lit.

### Procedure

The study protocol had Institutional Ethics approval (IEC/AP-13/2017). The ethics committee was informed regularly, as per protocol, about the progress of the study. During the study, informed consent was collected from each participant and/or their next of kin after explaining every detail pertaining to the study in a language that they understood clearly. First, the experimenter showed the experimental setup. The experimenter explained the tasks to each participant using a visual schedule. Also, the experimenter ensured that each participant understood the task before starting the task execution. A trained and qualified practicing psychiatrist in our team administered the ACE-III and MoCA scoring of the participants. Their demographic information was also collected. Then, the participant was asked to place his/her chin on the chin-rest. This was followed by the visual tasks being randomly presented to the participant. Each task started with an audio-visual presentation of how one needs to execute the task before our gaze-sensitive platform offered the task to the participant. This was followed by eye tracker calibration and task execution. The time required for the task completion was ∼5 min (including the time taken for task execution, audio-visual presentation of the task and calibration). This was the time taken by all the participants (while interacting with the gaze-sensitive platform) since this was predefined by the task design. The study required ∼25 min (comprising of introduction to the experimental setup, explanation of the tasks, ∼20 min of ACE-III and MoCA administration followed by ∼5 min for interaction with our gaze-sensitive platform) from each participant. While one took part in the tasks, the Task Computer stored his/her eye gaze data at the backend for subsequent offline analysis.

### Data processing

The gaze data acquired using the software development kit (sdk) that comes with the Intel RealSense camera for the eye tracking application was analyzed to extract oculomotor Performance indices, namely, Reaction Time (RT henceforth) for the HPS, VPS, HAS, and VAS tasks and the % Correct Hits for the MGF task.

### Extraction of Reaction Time

We computed the Reaction Time (RT) in terms of saccade latency (Polden et al., 2020) from the gaze data when one took part in the HPS, VPS, HAS and VAS tasks. The RT was computed from the temporal difference between the instant the Target appeared (t1) and the first relevant saccade was initiated (t2) using Eq. (1).


(1) $$R\,T=t_{2}-t_{1}$$

### Extraction of % Correct Hits

After one completed the MGF task, our system computed the % Correct Hits. For this, our gaze-sensitive platform computed the centroid of one’s fixation points. If the centroid (nearest to the Target) lied in the same quadrant as the Target, then the trial was labeled as having “Correct Hit,” else it was labeled as having “Incorrect Hit.” Finally, the % Correct Hits was calculated for all the 8 trials.

### Neuropsychological scoring used

In our present study, we used two Neuropsychological tests, namely, ACE-III (Weir et al., 2011) and MoCA (Lee et al., 2023). These tools have been reported as useful paper-and-pencil based cognitive instruments (Weir et al., 2011) and sensitive to early symptoms of cognitive decline (Galton et al., 2005).

### Clustering technique used

With an aim to identify the optimal combination of visual tasks that can be used to differentiate the participant groups, we performed clustering analysis in our study while using the oculomotor Performance indices. We chose the unsupervised clustering technique (instead of using supervised learning that fits a model adjusted to the labeled data) since we wanted to understand whether the oculomotor Performance indices were powerful enough to cluster the HC, MCI and D groups (irrespective of their labels being known) followed by evaluating the accuracy of the clustering with respect to the labels as obtained from the Neuropsychological tests. Specifically, for the unsupervised clustering technique, we used the K-means clustering. The K-means is well known clustering algorithm that has been used in clustering eye-movement data (Doherty et al., 2019). This algorithm clusters data points into k clusters depending on the mean of the centroids of the data points. The K-means considers the mean of the data while updating the cluster centroids (Doherty et al., 2019).

### Statistical analysis

We used SPSS version 20.0.0 (Field, 2009) for statistical analysis. The Shapiro–Wilk test of normality (Kim, 2014) showed that the oculomotor Performance indices were not normally distributed. Subsequently, for between-groups comparison, we performed independent sample non-parametric statistical test, namely, Wilcoxon Rank-Sum test (Kim, 2014). This test has been used by researchers dealing with eye tracking data (Bin Zahid et al., 2020).

## Results

The eye tracking data acquired during the HPS, VPS, HAS, VAS, and MGF tasks were processed to compute one’s oculomotor Performance indices, namely, RT (for HPS, VPS, HAS, and VAS tasks) and % Correct Hits (for MGF task). Subsequently, these indices were used to identify the visual tasks from among the HPS, VPS, HAS, VAS, and MGF tasks that can differentiate the HC, MCI, and D groups [labeled using the Neuropsychological tests (Table 1)]. Also, we investigated the relation between such indices (for each of the visual tasks) and the Neuropsychological test scores. In addition, we wanted to understand whether the visual tasks in isolation or in combination can offer optimal solution while clustering the participants belonging to the three groups. Finally, we wanted to ensure that the variations in the task-specific oculomotor behavior (related to the deterioration in one’s cognitive performance) were not affected by the varying demographics of the participants.

### Identifying visual tasks that can differentiate the participant groups using oculomotor behavior

To identify the visual tasks from among the HPS, VPS, HAS, VAS, and MGF tasks that can differentiate the HC, MCI, and D groups, we analyzed the oculomotor Performance indices (namely, RT and % Correct Hits) of the participant groups corresponding to each task.

**FIGURE 4:**
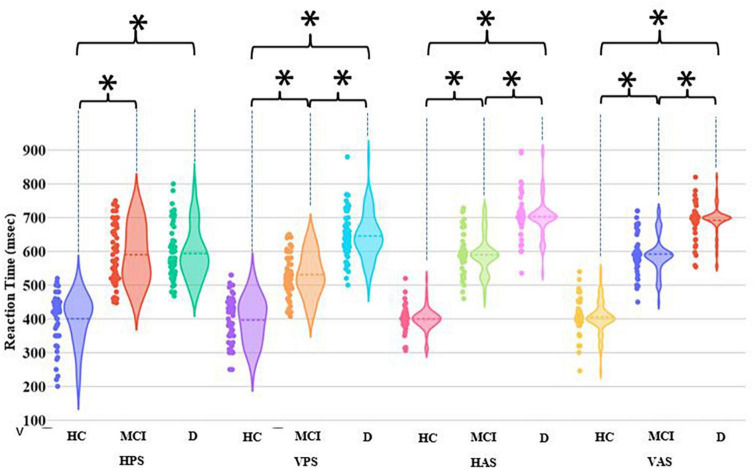
Comparative group analysis of Reaction Time for different tasks. [*–Statistical significance (*p*-value < 0.01); HPS, Horizontal Pro-saccade; VPS, Vertical Pro-saccade; HAS, Horizontal Anti-saccade; VAS, Vertical Anti-saccade].

Let us first consider the HPS, VPS, HAS, and VAS tasks. For the HC group, the group average RT remained nearly the same irrespective of the tasks (Figure 4). Both MCI and D groups had statistically higher (*p*-value < 0.01) group average RT than the HC group across all the four tasks. This infers that the individuals belonging to the MCI and D groups took more time in initiating their relevant saccade before fixating on the Target than their healthy aging counterpart. Also, the MCI and D groups were found to be statistically different (*p*-value < 0.01) in terms of their RT values for VPS, HAS, and VAS tasks. Though each of the four visual tasks could statistically differentiate each of the MCI and D groups from the HC group and also differentiate the MCI group from the D group (except the HPS task), yet, from a closer look at the violin graph, we can see that only for the HAS and VAS tasks, most of the RT values are concentrated around the respective mean RT values with limited dispersion (unlike that in the case of HPS and VPS tasks).

**FIGURE 5:**
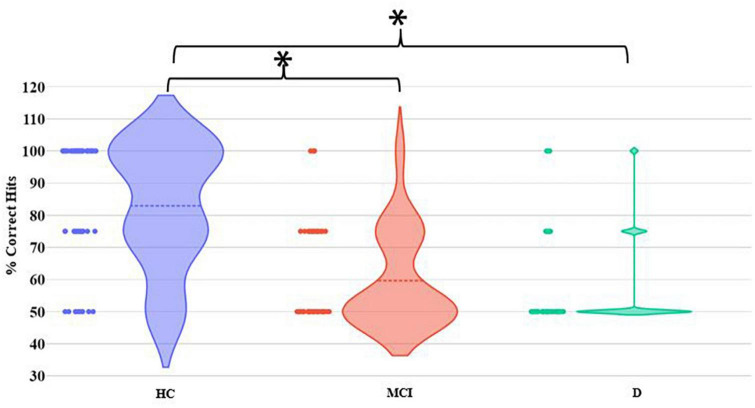
Comparative group analysis of % Correct Hits for MGF Task. [*–Statistical difference (*p*-value < 0.01)].

Let us now consider the MGF task. The MCI and D groups were found to demonstrate statistically (*p*-value < 0.01) different group average % Correct Hits from the HC group (Figure 5). In contrast, there existed no statistical difference in the group average % Correct Hits between the MCI and D groups.

### Understanding the relation between the visual task-specific oculomotor performance indices with the Neuropsychological test scores

We wanted to understand the relevance of the oculomotor Performance indices during each visual task with regard to the Neuropsychological test scores. For this we computed the Spearman’s Correlation (Zar, 2005) of RT (for HPS, VPS, HAS, and VAS tasks) and % Correct Hits (for MGF task), with the ACE-III and MoCA scores (Table 1).

**FIGURE 6:**
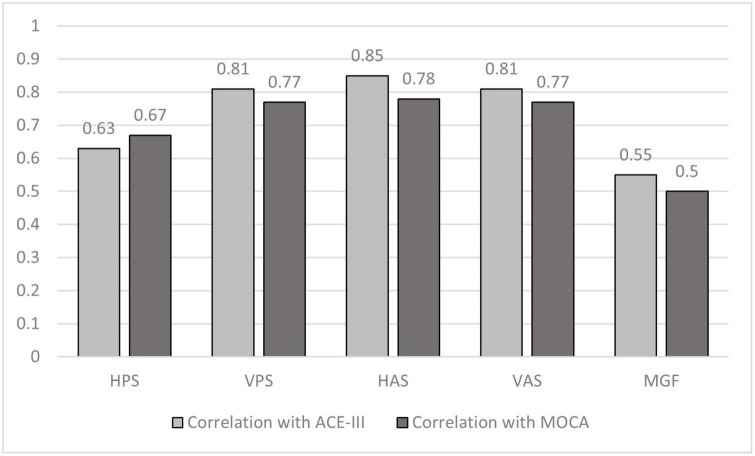
Comparative group analysis of correlation coefficient of ACE–III scoring and MoCA scoring with Reaction Time for different tasks. HPS, Horizontal Pro-saccade; VPS, Vertical Pro-saccade; HAS, Horizontal Anti-saccade; VAS, Vertical Anti-saccade.

Our results (Figure 6) indicate that the saccade latency corresponding to the VPS, HAS, and VAS tasks strongly correlated with correlation coefficients being >0.7 (Akoglu, 2018) (as evident from the absolute correlation coefficient) with both the ACE-III and MoCA scores with latency corresponding to the HAS and VAS being more correlated to both the Neuropsychological test scores closely followed by the latency for the VPS task. In addition, moderate correlation was observed for the HPS and MGF tasks.

### Identification of the optimal combination of visual tasks for clustering of HC, MCI, and D groups based on the oculomotor performance indices

With the saccade latency corresponding to the VPS, HAS, and VAS tasks emerging as being strongly correlated (Akoglu, 2018) with the Neuropsychological tests, we wanted to understand whether the VPS, HAS, and VAS tasks in isolation or in combination can offer optimal solutions for classification of the HC, MCI, and D groups (as labeled based on the Neuropsychological test scores). We employed an unsupervised clustering technique, namely, K-means clustering (“Materials and methods”). While considering the values of RT during the VPS, HAS, and VAS tasks, we obtained seven configurations of the input vector (Conf_1_ to Conf_7_ henceforth) applied to the clustering algorithm based on varying combinations of the visual tasks. In addition, though the HPS and MGF tasks were not strongly correlated with the Neuropsychological test scores, we also extended oculomotor Performance indices for HPS and MGF tasks in the classification for the sake of comprehensive comparison. While considering the values of RT and the % Correct Hits during the HPS and MGF tasks, respectively and combining the oculomotor indices with the RT values for VPS, HAS, VAS tasks [along with normalizing all the oculomotor indices on a scale of 0–1 using min-max normalization (Weijun and Zhenyu, 2011)], we obtained twenty-four configurations of the input vector (Conf_8_ to Conf_31_ henceforth) to be applied to the clustering algorithm. The classification accuracy for the HC, MCI and D groups for each of the configurations is presented in Table 2 (with the maximum classification accuracy for each configuration being presented in bold).

**TABLE 2:** Clustering accuracy.

Configuration	Test (s)	% Correctly clustered in HC group	% Correctly clustered in MCI group	% Correctly clustered in D group
Conf_1_	VPS and HAS	**98.3**	86.6	88.3
Conf_2_	VPS and VAS	**96.6**	93.3	76.6
Conf_3_	HAS and VAS	**100**	91.6	93.3
Conf_4_	VPS, HAS, and VAS	**98.3**	96.6	91.6
Conf_5_	VPS	51.67	68.33	**83.33**
Conf_6_	HAS	**88.33**	83.33	83.33
Conf_7_	VAS	85	80	**86.6**
Conf_8_	HPS	**68.33**	53.33	56.67
Conf_9_	MGS	**83.33**	66.67	76.67
Conf_10_	HPS and VPS	**100**	50	65
Conf_11_	HPS and HAS	**96.7**	70	83.3
Conf_12_	HPS and VAS	**100**	53.3	75
Conf_13_	HPS and MGF	**88.33**	56.7	73.3
Conf_14_	MGF and VPS	51.67	68.3	**83.3**
Conf_15_	MGF and HAS	**98.33**	80	86.7
Conf_16_	MGF and VAS	**95**	80	86.7
Conf_17_	HPS, VPS, and HAS	**100.0**	55.0	70.0
Conf_18_	HPS, VPS, and VAS	**98.3**	85.0	90.0
Conf_19_	HPS, VPS, and MGS	**98.3**	50.0	68.3
Conf_20_	HPS, HAS, and VAS	**100.0**	86.7	93.3
Conf_21_	HPS, HAS, and MGS	**100.0**	58.3	53.3
Conf_22_	HPS, VAS, and MGS	**100.0**	53.3	75.0
Conf_23_	VPS, HAS, and VAS	**98.3**	96.7	91.7
Conf_24_	VPS, HAS, and MGS	**96.7**	93.3	76.7
Conf_25_	VPS, VAS, and MGS	**98.3**	86.7	90.0
Conf_26_	HPS, VPS, HAS, and VAS	**98.3**	96.7	91.7
Conf_27_	HPS, VPS, HAS, and MGS	**100.0**	95.0	75.0
Conf_28_	HPS, VPS, VAS, and MGS	**98.3**	85.0	90.0
Conf_29_	HPS, HAS, VAS, and MGS	**100.0**	86.7	93.3
Conf_30_	VPS, HAS, VAS, and MGS	**98.3**	96.7	91.7
Conf_31_	HPS, HAS, VPS, VAS, and MGS	**98.3**	96.7	91.7

HC, healthy aging control; MCI, mild cognitive impairment; D, advanced stage of cognitive decline. Bold values indicate the maximum values of clustering accuracy (corresponding to a particular group) for a particular combination of oculomotor Performance indices.

It can be seen from Table 2 that while considering combinations of oculomotor Performance indices of any two of the visual tasks, we could achieve a maximum of at least ∼92% of clustering accuracy for Conf_3_ while segregating the HC, MCI and D groups based on the values of RT. Likewise, while considering combinations of oculomotor Performance indices of any three of the visual tasks, a maximum of at least ∼92% of clustering accuracy was achieved for Conf_4_. Again, while considering combinations of oculomotor Performance indices of any four of the visual tasks, a maximum of at least ∼92% of clustering accuracy was achieved for Conf_26_ and Conf_30_. Also, a maximum of at least ∼92% clustering accuracy was obtained while considering combination of oculomotor Performance indices of all the five visual tasks (Conf_31_).

While considering the values of RT and % Correct Hits as applicable for each of the visual tasks in isolation, we found that for Conf_5_, Conf_6_, Conf_7_, Conf_8_, Conf_9_ (Table 2), there was at least ∼52%, ∼83%, ∼80%, 68%, and 83%, respectively of clustering accuracy while segregating the HC, MCI and D groups.

**FIGURE 7:**
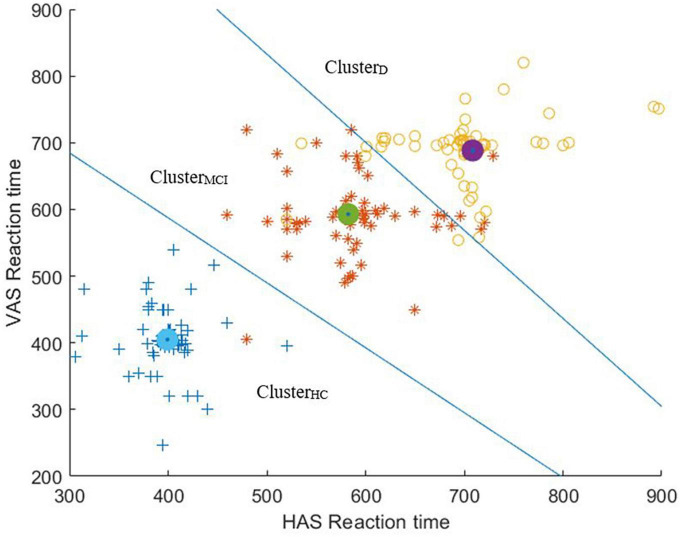
Scatter plot of Reaction Times of HAS and VAS overlaid with the clustering output (expressed in terms of Voronoi diagram).

To summarize, given that a combination of two visual tasks (true for Conf_3_) was found as the optimal configuration which could give us a maximum of at least ∼92% of clustering accuracy while segregating the HC, MCI and D groups, here we present the scatter plot overlaid with the clustering output [expressed in terms of Voronoi diagram (Erwig, 2000)] for Conf_3_ (Figure 7). It can be seen from Figure 7 that the number of misclassifications is minimal for Conf_3_.

### Understanding the effect of demographics on visual task-specific oculomotor behavior

In the process of working with one’s task-specific oculomotor Performance indices that are sensitive to one’s cognitive performance, we wanted to further explore the effect of participant demographics on the oculomotor Performance indices corresponding to each visual task. For this, we categorized the participants (belonging to HC, MCI, and D groups who were not statistically different with respect to age with most of them being less than 60 years of age, based on their availability during enrolment from a community center that maintained this cohort) based on (i) gender i.e., male and female and (ii) education, such as those with “Primary” and “Junior college and above” education levels (with up to 4 years and >10 years of education, respectively). With the VPS, HAS, and VAS tasks emerging as being strongly correlated with the Neuropsychological tests and achieving at least ∼92% of clustering accuracy, we chose to study the oculomotor behavior for these three tasks in the perspective of demographics. It can be seen from Tables 3, 4 that the values of RT during each of VPS, HAS, and VAS tasks of the participants (for each of HC, MCI, and D groups) segregated based on gender and education were nearly similar without any statistical difference. From this, we can infer that the variations in the participants’ demographics did not have any significant effect on the task-specific oculomotor behavior.

**TABLE 3:** Comparative analysis of RT and % Correct Hits between male and female participant group.

Mean (Standard error)						
		HPS (ms)	VPS (ms)	HAS (ms)	VAS (ms)	MGF (%)
HC	M	409.9 (12.2)	383.5 (14.6)	401.0 (18.5)	396.7 (22.5)	84.0 (2.8)
	F	378.4 (41.6)	444.1 (33.8)	424.0 (52.2)	412.1 (27.1)	82.0 (7.0)
	Sig	ns	ns	ns	ns	ns
MCI	M	569.4 (13.3)	546.3 (10.6)	583.7 (15.0)	604.7 (19.0)	59.4 (2.8)
	F	551.3 (17.2)	517.7 (18.2)	580.3 (23.5)	569.5 (10.9)	59.6 (5.3)
	Sig	ns	ns	ns	ns	ns
D	M	593.4 (19.6)	676.3 (32.2)	705.0 (42.4)	693.1 (34.5)	55.7 (2.8)
	F	583.7 (13.8)	610.2 (25.3)	705.1 (30.0)	674.9 (27.6)	61.0 (4.1)
	Sig	ns	ns	ns	ns	ns

“ns”: *p*-value > 0.05; HC, healthy aging control; MCI, mild cognitive impairment; D, advanced stage of cognitive decline; M, male; F, female.

**TABLE 4:** Comparative analysis of RT and % Correct Hits between participants with “Junior college and above” and “Primary” education.

Mean (Standard error)						
		HPS (ms)	VPS (ms)	HAS (ms)	VAS (ms)	MGF (%)
HC	JCA	403.8 (13.7)	383.1 (15.0)	402.1 (18.6)	404.5 (18.2)	85.5 (2.8)
	P	419.1 (18.6)	474.6 (42.5)	435.0 (15.0)	333.0 (87.0)	77.5 (5.8)
	Sig	ns	ns	ns	ns	ns
MCI	JCA	590.6 (17.6)	544.6 (13.6)	586.1 (16.6)	579.0 (18.6)	49.4 (0.6)
	P	591.5 (51.9)	520.5 (9.9)	532.5 (12.5)	685.0 (35.0)	45.0 (5.0)
	Sig	ns	ns	ns	ns	ns
D	JCA	605.7 (24.4)	634.8 (20.7)	693.9 (22.4)	729.6 (37.7)	60.0 (3.7)
	P	567.9 (27.9)	586.9 (9.9)	613.8 (35.7)	690.4 (65.5)	54.0 (2.0)
	Sig	ns	ns	ns	ns	ns

“ns”: *p*-value > 0.05; JCA, Junior college and above; P, primary; Sig, significance; HC, healthy aging control; MCI, mild cognitive impairment; D, advanced stage of cognitive decline.

## Discussion

In this work, we have investigated visual task-specific oculomotor behavior (of a group of cognitively unimpaired healthy aging individuals, a group with MCI and a group with dementia). These groups were labeled using two standard Neuropsychological tests. While the participants took part in Anti-saccade (HAS and VAS), Pro-saccade (HPS and VPS) and Memory Guided Fixation (MGF) visual tasks, their oculomotor behavior was evaluated in terms of oculomotor Performance indices [e.g., Reaction Time (RT, i.e., saccade latency) and memory-guided spatial fixation accuracy (% Correct Hits)]. The oculomotor Performance indices during the Anti-saccade tasks could differentiate individuals with MCI and dementia from their cognitively unimpaired healthy aging counterparts with good clustering accuracy. Also, the oculomotor Performance indices during the Anti-saccade tasks were found to have strong correlation with both the Neuropsychological test scores.

### Visual tasks differentiating the HC, MCI, and D groups using oculomotor behavior: importance of Anti-saccade tasks

In a bid to identify the visual tasks that can differentiate the participant groups, we carried out comparative analysis of their oculomotor Performance indices corresponding to the HPS, VPS, HAS, VAS, and MGF tasks. All of the visual tasks were found to be capable of statistically differentiating the MCI and D groups from the HC group. However, one of the two remarkable findings of this work was that three of the five visual tasks (namely VPS, HAS, and VAS) could also statistically differentiate the MCI group from the D group based on the saccade latency. In addition, the other finding was that for the Anti-saccade Tasks (i.e., the HAS and VAS tasks) differentiating the MCI group from the D group, the dispersion in the values of RT was lesser than that in the case of Pro-saccade tasks. This might indicate that the effect of the heterogeneity in the cognitive performance within each of HC, MCI and D groups causing the dispersion in the values of RT [as evident from the violin graph (Figure 4)] was lesser for the Anti-saccade tasks than for the Pro-saccade tasks. Such an observation on the HAS and VAS tasks (out of the three tasks, i.e., VPS, HAS, and VAS) might be indicative of the task of inhibiting the reflexive saccade (15; during the Anti-saccade task) being more sensitive to the cognitive performance than making a saccade in the vertical direction [i.e., VPS task without any inhibitory control (Hutton, 2008)] as hypothesized, at least in our participant sample. One of the probable reasons behind Anti-saccade task emerging as powerful in differentiating the MCI group from the D group (in addition to differentiating MCI and D groups from HC group) can be due to the higher-level voluntary control involving extensive cortical areas (Hallett, 1978) being true for the Anti-saccade tasks. The other possible reason being inability to resolve conflict between volitional and reflexive saccades (applicable for the Anti-saccade tasks) is one of the key hallmarks of cognitive deficit (Hutton and Ettinger, 2006) characterizing the MCI and D groups.

### Optimal combination of visual tasks for clustering participant groups based on the oculomotor performance indices: HAS and VAS tasks offering complementary information

One of our objectives was to identify the optimal combination of the visual tasks that can cluster the HC, MCI and D groups based on the saccade latencies. Different investigators have used various visual tasks to cluster or classify individuals having varying levels of cognitive impairment while using oculomotor data. For example, Oyama et al. (2019) have used moving shapes as visual stimuli while classifying individuals having MCI and dementia (with Area Under the Curve of 0.84) based on oculomotor data. Also, investigators (Nie et al., 2020) have used various visual stimuli consisting of complex patterns, familiar and novel images while classifying MCI and the cognitively healthy aging counterparts and reported of achieving an accuracy of 70%. Some researchers have used various biomarkers to classify MCI from their cognitively healthy aging counterparts such as Jiang et al. (2019) who used electroencephalogram and oculomotor data while using dynamic visual stimuli while achieving an accuracy of ∼80%. Further, Mengoudi et al. (2020) have used scene-based images as visual stimuli to classify individuals with dementia and cognitively healthy aging individuals based on oculomotor data with reported accuracy being ∼78%. Though powerful, none of these studies (to the best of our knowledge) have investigated varying combination of such visual tasks that can offer optimal solutions. Having understood that the saccade latencies of our participant groups corresponding to the VPS, HAS, and VAS tasks were strongly correlated with the Neuropsychological test scores, we wanted to identify the optimal combination of the visual tasks that can cluster the HC, MCI, and D groups. Our results indicated that a combination of HAS and VAS tasks (Conf_3_) fed to the K-means clustering algorithm offered the clustering accuracy similar to other combinations having three tasks (i.e., Conf_4_), four tasks (i.e., Conf_26_ and Conf_30_), and five tasks (i.e., Conf_31_) with a tie of a minimum accuracy of ∼92%. This suggests that the combination of HAS and VAS tasks can offer an optimal clustering performance for the HC, MCI, and D groups that might indicate possible complementarity of the HAS and VAS tasks.

### Limitations

Although our results are promising, there are certain limitations. One of the limitations is that in our present study, we investigated only some of the oculomotor indices (while attempting to make our application less time-intensive) and it might be useful to include more oculomotor indices (for example the Anti-saccade error for Anti-saccade tasks performed incorrectly) in future that can possibly contribute to strengthening the clustering accuracy. Additionally, we did not investigate other biomarkers such as electroencephalogram (Jiang et al., 2019), peripheral physiological indices (Perugia et al., 2017), e.g., electrodermal activity, heart rate, etc. This is because these indices can offer valuable markers to one’s cognitive health (Perugia et al., 2017; Chai et al., 2023). Though our present study was used to identify the optimal combination of visual tasks that can cluster the participants into different participant groups (irrespective of the clinical classification) based on a limited set of oculomotor performance indices in response to the five visual tasks, in future, we plan to explore different biomarkers while participants undertake the various visual tasks (with no intention to use our platform for diagnostic purposes). Another limitation was the use of only black-on-white (i.e., black colored stimulus on a white colored background screen) for our visual stimulus presentation. Though research shows that one’s oculomotor indices are not affected by the black-on-white or white-on-black (i.e., white colored stimulus on a black colored background screen) visual stimulus (Pratt and Trottier, 2005) yet our present study can be extended to include both variations of visual stimulus of the tasks in the future. Another limitation was the limited number of trials for each visual task for each participant, though we restricted the number of trials keeping in mind that the setup would be used mostly by elderly individuals who might find it difficult to keep their chin on the chin-rest over a larger number of trials. However, in future, we plan to carry out extended study to identify the optimum number of trials in each task that might work well for elderly individuals and also contribute to improved classification. Given that the age of our participants belonging to HC, MCI, and D groups had no statistical difference, we did not consider effect of age while analyzing the effect of demographics on visual task-specific oculomotor behavior. Given the fact that there exists strong relationship of age with the risk for MCI and dementia (van der Flier and Scheltens, 2005) further investigation can be done in future to analyze the effect of age on the oculomotor behavior of individuals with healthy aging, MCI and cases of dementia. Again, the cohort at our study site had a considerable number of relatively younger individuals with MCI and dementia who were recruited in our study based on convenience sampling, it might be that our study sample was not completely representative of the population which could be one of the limitations of the convenience sampling used in our study. In addition, though our present unsupervised clustering approach applied on the oculomotor Performance indices did not use the labels given to the participant pool who was screened based on the reports of their functional abilities, any change in cognition recognized by the affected individual or observers along with objective impairment in one or more cognitive domains as obtained using the ACE III and MOCA, it needs to be noted that the ACE III and MOCA are screening tools. Although, these screening tools have been reported in literature to have good diagnostic accuracy (Matias-Guiu et al., 2017; Bruno and Schurmann Vignaga, 2019) with ACE III has been validated for diagnosing dementia using standardized Neuropsychological tests (Mekala et al., 2020), we plan to extend our present research while using other Neuropsychological diagnostic tests in the future. Also, further research is required to carry out deeper exploration of the various aspects of gaze-based performance data while identifying different types of dementia (Fronto-temporal Dementia/Vascular Dementia). This might need access to brain imaging data that can help isolate the affected brain regions and its relation with task-specific oculomotor behavior.

## Conclusion and future works

In this work, we have described visual tasks that were used to differentiate HC, MCI, and D groups based on oculomotor Performance indices, namely, RT (for HPS, VPS, HAS, and VAS tasks) and % Correct Hits (for MGF task). Also, we presented our findings on the correlation of the task-specific oculomotor Performance indices with Neuropsychological test scores. Finally, we offered our observations on possible optimal combination of the visual tasks that can effectively cluster the HC, MCI, and D groups based on the saccade latencies. Last, but not the least, we could show that the variations in the task-specific oculomotor behavior of the participants were not significantly affected by their varying demographics. Though our results are promising, in future we would like to extend our study to investigate the potential of the Anti-saccade tasks to characterize cases of Fronto-temporal dementia, Alzheimer’s Disease, etc.

## Data availability statement

The raw data supporting the conclusions of this article will be made available by the authors, without undue reservation.

## Ethics statement

The studies involving human participants were reviewed and approved by the Institute Ethics Committee, Indian Institute of Technology, Gandhinagar. The patients/participants provided their written informed consent to participate in this study.

## Author contributions

DR and UL drafted the manuscript and it was shared among the other co-authors for their inputs. DR contributed to the data analysis and was assisted by DD. UL supervised the technical implementation and data analysis. AlD contributed to the enrollment of participants and assessment of clinical measures. AbD and AnD offered the clinical inputs. All authors contributed to the article and approved the submitted version.
